# Evaluation of ENA-6 Profile by ELISA Immunoassay in Patients with Systemic Lupus Erythematodes

**DOI:** 10.1155/2012/321614

**Published:** 2012-10-11

**Authors:** Izeta Aganovic-Musinovic, Jasenko Karamehic, Lamija Zecevic, Faris Gavrankapetanovic, Nesina Avdagic, Asija Zaciragic, Tomislav Jukic, Nerima Grcic, Suvada Svrakic

**Affiliations:** ^1^Center for Genetics, Medical Faculty, University of Sarajevo, Cekalusa 90, Sarajevo, Bosnia and Herzegovina; ^2^Department of Clinical immunology, KCUS, Sarajevo, Bosnia and Herzegovina; ^3^Department of Biomedicine and Public Health, Faculty of Medicine, Osijek University, Osijek, Croatia

## Abstract

Autoimmune diseases occur in 3−5% of the population. Study included 30 patients with clinically diagnosed SLE and 30 healthy controls (American college of Rheumatology, 1997). SLE was diagnosed according to criteria issued in 1997 by the American College of Rheumatology (ACR). The aim of this study was to evaluate concentration values of each antigen of ENA-6 profile in SLE, to investigate possible correlation between the concentration of Sm antibodies and CIC, and to test their use as possible immunobiological markers in SLE. Furthermore, the aim of our study was to determine whether there is a correlation between Sm antibodies and CIC and SLE activity. The results revealed that all of these ENA-6 and Sm antibodies as biomarkers complement diagnoses of active SLE but their use as solo markers does not allow classifying patients with SLE. Our study has shown that based on calculations from ROC curves, Sm/RNP was clearly a very important marker for diagnosis of SLE (cut off ≥ 9.56 EU, AUC 0,942). The high incidence of Scl-70 (10%) reactivity suggests that ELISA monitoring of this antibody produces more false positive results than other multiplex assay. An important conclusion that can be drawn from the results of our study is that laboratory tests are no more effective than clinical examination for detecting disease relapse, but are helpful in the confirmation of SLE activity.

## 1. Introduction

 Autoimmune diseases occur in 3–5% of the population [1], as a result from myriad of genetic and environmental factors that lead to altered immune reactivity [2, 3]. The alterations in the immune system initiated by a loss of immunological tolerance to self-antigens lead to the development of autoreactive phenomena that can be detected in the peripheral blood. Defining specific pathogenic mediators that may trigger the development or progression of an autoimmune disease remains a focus of intense research. 

Systemic lupus erythematosus (SLE) is an autoimmune disease characterized by B cell hyperactivity resulting in overproduction of autoantibodies against cytoplasmic, nuclear, and surface antigens and immune complex formation [4, 5]. 

The majority of autoantibodies found in SLE are targeted at intracellular nucleoprotein particles. 98% of patients have antinuclear antibodies and antidouble-stranded DNA antibodies are found in 50–80% of patients [6]. These autoantibodies are frequently targeted against intracellular antigens of the cell nucleus (double- and single- stranded DNA (dsDNA and ssDNA, resp.,) histones, and extractable nuclear antigens (ENAs). Most of these autoantibodies are not specific for SLE and might be produced nonspecifically as a result of polyclonal B cell activation [7, 8]. ENA-6-Profile is useful for the diagnosis of systemic autoimmune rheumatic diseases such as systemic lupus SLE, Sjögren's syndrome, Sharp syndrome, polymyositis/dermatomyositis, or progressive systemic scleroderma (PSS) [9]. Because antibodies against ENA have a partial marker function for the individual diseases, the isolated detection of these antibodies with the ENA-6-profile allows serological differentiation of these diseases. 

Autoimmune disease detection protocol starts with determination of ANA (antinuclear antigen). Positive ANA test leads to further investigation of extractible nuclear antigens (ENA) [10].

The prevalence (70%) of anti-dsDNA autoantibody is much higher in SLE, giving a higher diagnostic sensitivity than the similarly disease-specific anti-Sm autoantibodies (30%). In some pathological conditions, like SLE, the concentration level of circulating immune complexes (CIC) increases in tissues and causes the activation of humoral immunity effectors' mechanisms, such as of classical complement pathway activation [11]. 

Six of the most diagnostically useful autoantibodies include those to Ro (SSA), which are found in 40 to 60% of patients with Sjögren's syndrome and in 25 to 35% of patients with ANA-positive SLE. La (SSB) autoantibodies are found in 50 to 60% of patients with Sjögren's syndrome and 5 to 15% of SLE patients [12].

Smith (Sm) antibodies are highly specific for SLE but only occur in 30 to 35% of cases [13]. ELISA monitoring of extractible antinuclear antibodies—Smith antigen is usually used with the concentration value of ds-DNA to control the disease activity [14].

Antibodies to ribonucleoprotein (RNP) are found in 95 to 100% of patients with Mixed Connective Tissue Disease (MCTD) but are also found in up to 45% of patients with SLE [15]. The presence of anti-RNP antibody alone strongly suggests a diagnosis of MCTD [16]. Scleroderma (Scl-70) autoantibodies are the specific markers for scleroderma and are found in up to 60% of patients diagnosed with this disorder [17]. The Jo-1 autoantibody is one of a family of characteristic autoantibodies seen in myositis patients [18, 19].

The aim of this study was to evaluate concentration values of each antibody of ENA-6 profile in SLE, to investigate possible correlation between the concentration of Sm antibody and CIC and to test their use as possible immunobiological markers in SLE. Furthermore, the aim of our study was to determine whether there is a correlation between Sm antibody and CIC and SLE activity.

## 2. Material and Methods

### 2.1. Patients

Study included 30 serum samples submitted to our reference laboratory for autoimmune testing from patients with diagnosed SLE diseases and 30 serum samples from healthy individuals. Patients were recruited through input clinical diagnosis according to criteria of SLE diagnosis issued in 1997 by American College of Rheumatology [20]. 

### 2.2. ENA-Assay

The detection of anti-nuclear antibodies (ANA's) has long been an important tool in the diagnosis of systemic rheumatic diseases. The antigens used in their detection are purified by the saline extraction of human or animal nuclei, and this has led them being termed as extractable nuclear antigens (ENA's). The most commonly measured ENA specifications are anti-SS-A/Ro, anti-SS-B/La, anti-Sm, anti-Sm/RNP, anti-Jo-1, and anti-Scl-70 [21]. 

To determine concentrations of Sm-antibody and other ENA 6 antibodies as well as CIC, we have used ELISA method [22].

### 2.3. Principle of the Procedure

The Autostat II assay is a solid phase immunosorbent assay (ELISA) in which the analyte is indicated by a color reaction of an enzyme and substrate. The Autostat II wells are coated with purified antigens. The device used was Hytec 288.

On adding diluted serum to the wells the antibodies bind to the antigens. After incubating at room temperature and washing away unbound material, horseradish peroxidase conjugated anti-IgG monoclonal antibody was added, which binds to the immobilized antibodies.

Following further incubation and washing, tetra-methyl benzidine substrate (TMB) is added to each well. The presence of the At-Ag complex turns the substrate to a dark blue color. Addition of the stop solution turns the color to yellow.

The color intensity is proportional to the amount of autoantigens present in the original serum sample [23–25].

### 2.4. Interpretation of Results

For antigens of ENA 6 results below 10 are considered negative, while the results above 15 are considered positive. Results in the range among 10 to 15 are considered equivocal [26, 27]. Referent intervals are as shown in Table 1 [28].

For dsDNA both techniques were applied for determination, ELISA, and immunofluorescence assay (IFA). The basic principle of the procedure is based on the use of slides with epithelial cells (Hep-2 cells) as substrates that are incubated in few steps with diluted serum. The unbound material is removed by aspirating and washing. The drop of the fluorescence conjugate (anti-human IgG fluorescein labeled containing blue dye and 0.099 sodium azid) is added [29]. Depending on the amounts of autoantibodies in specimens, using IF microscope, it is possible to detect different intensity degree of apple-green fluorescence light. Fluorescence grade is determined as 5+; 4+; 3+; 2+, and positive and negative as zero [30].

### 2.5. Statistical Analysis

The Kolmogorov-Smirnov test of normality was used to test the distribution of variables. Since all variables were skewed they are presented as median and interquartile ranges. Mann-Whitey *U*-test was used to compare differences between two groups. Since all variables were highly skewed, correlations were assessed by Spearman's test. A *P* value of <0.05 was considered statistically significant [31].

Sensitivity, specificity, positive, and negative predictive value were calculated according to the following formula [32]:

(1) Sensitivity=a(a+c),Specificity=d(b+d),Positive  predictive  value=a(a+b),Negative  predictive  value=d(c+d),

where *a* = true-positive cases, *b* = false-positive cases, *c* = false-negative cases, and *d* = true-negative cases [32].

Receiver operating characteristic (ROC) curves were constructed by calculating the sensitivities and specificities of ENA 6 SS-A, ENA6 SS-B, ENA6 Sm, Sm/RNP, Jo-1, or SCL 70 assays at several cut-off points [33–35].

The software used was SPSS for Windows (version 17.0; SPSS, Chicago, IL, USA). 

## 3. Results

**Figure 1:**
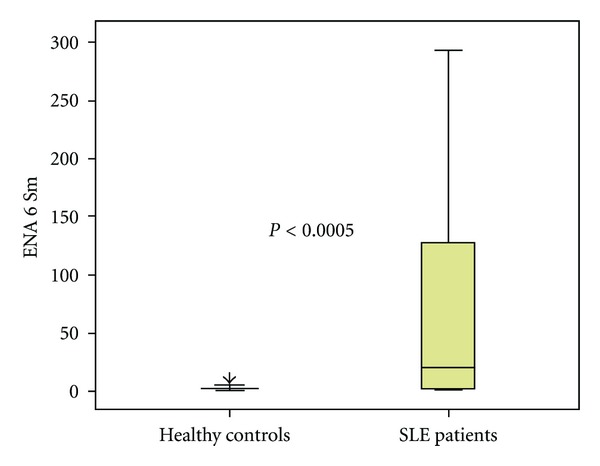
Serum ENA6 Sm concentration in healthy controls and SLE patients. Each bar shows upper and lower quartile, while the square and its central bar indicate interquartile range and median.

Figure 1 shows the median and interquartile range of ENA6 Sm serum concentration in the healthy subjects (1, 65; 0, 60–2, 62) and in the SLE patients (19, 07; 1, 97–130,44). Serum ENA6 Sm antibody concentrations in SLE patients were significantly higher compared to healthy controls (*P* < 0.0005).

**Figure 2:**
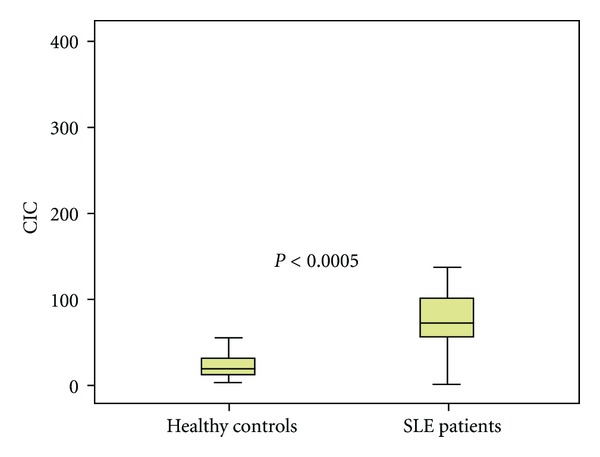
Serum CIC concentration in healthy controls and SLE patients. Each bar shows upper and lower quartile, while the square and its central bar indicate interquartile range and median.

Figure 2 shows the median and interquartile range of circulating immune complexes (CIC) serum concentration in healthy subjects (19,00; 12,00–32,00) and in the SLE patients (71,14; 52,99–102,04). Serum CIC concentrations in SLE patients were significantly higher compared to healthy controls (*P* < 0.0005). 

**Figure 3:**
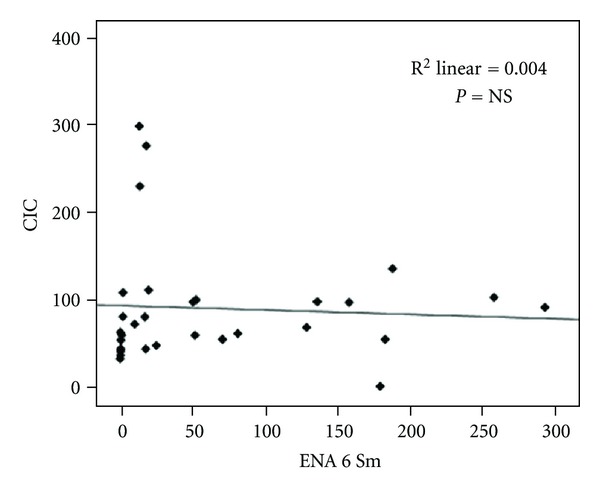
Spearman's correlation analysis between ENA6 Sm and CIC.

Results did not show significant correlation between ENA6 Sm and CIC (*r* = 0.29; *P* = NS) (Figure 3).

**Figure 4:**
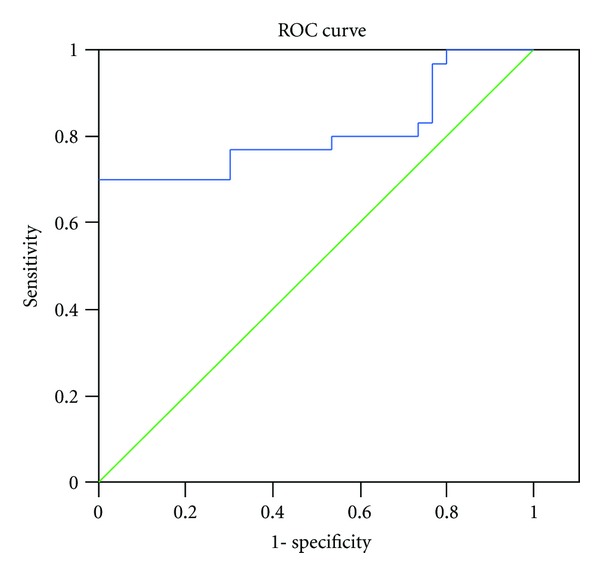
Receiver operating characteristic (ROC) curve of ENA6 Sm for differentiation between SLE patients and healthy control.

**Figure 5:**
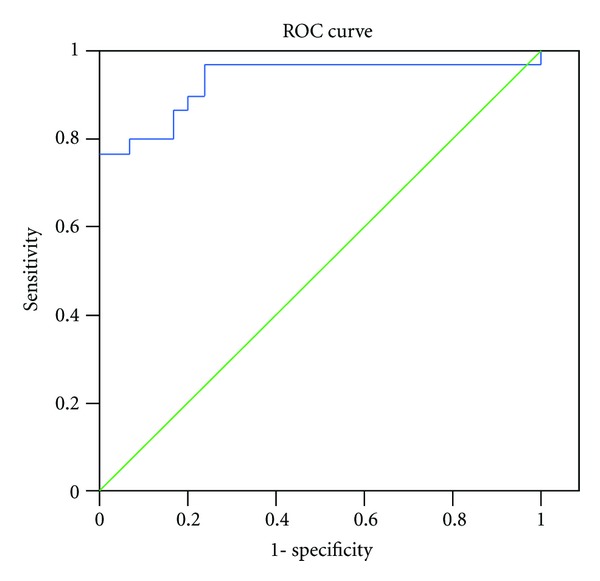
Receiver operating characteristic (ROC) curve of circulating immune complexes (CIC) for differentiation between SLE patients and healthy control.

The ROC curves for ENA6 Sm and CIC in the patients with SLE and healthy controls are shown in Figures 4 and 5. 

**Figure 6:**
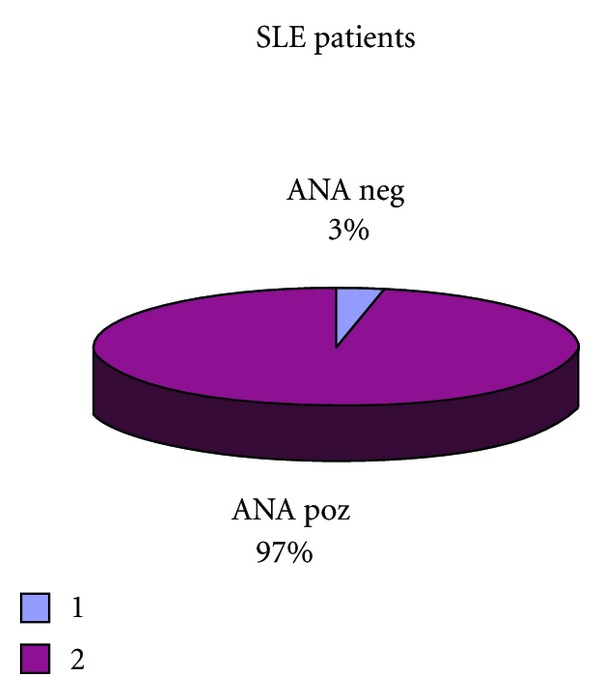
It presents number patients of positive and negative ANA testing's.

In our study sample 97% of patients were ANA positive and 3% were ANA negative as presented at Figure 6. 

**Figure 7:**
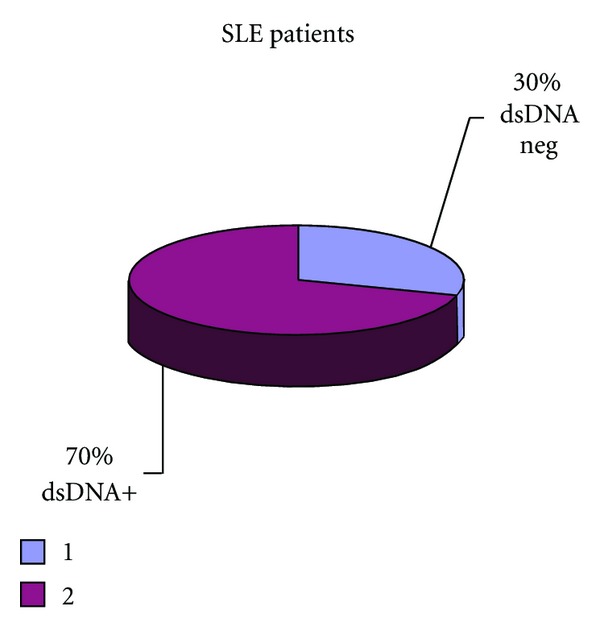
Percentage of positive and negative dsDNA patients.

Figure 7 presents that 30% of patients were dsDNA negative and 70% were dsDNA positive.

Based on the proposed cut-off values, the sensitivity, and specificity of the ENA6 Sm and CIC were calculated. 

Table 2 shows the predictive power of each marker in distinguishing patients with SLE and healthy controls.

Serum concentration of ENA6 SS-A in the SLE patients (11.70; 2.85–183.51) was significantly higher (*P* < 0.001) compared to healthy controls (3.55; 1.20–5.75). Serum concentration of ENA6 SS-B in the SLE patients (6.64; 1.71–46.32) was significantly higher (*P* < 0.01) if compared to healthy controls (3.10; 1.45–5.90). Serum concentration of ENA6 Sm in the SLE patients (19.93; 2.27–135.95) was significantly higher (*P* < 0.0005) compared to healthy controls (1.65; 0.60–2.62). Serum concentration of Sm/RNP in the SLE patients (56.61; 17.70–166.96) was significantly higher (*P* < 0.0005) compared to healthy controls 1.20 (0.50–2.80). Serum concentration of Jo-1 in the SLE patients (2.22; 1.40–4.79) was significantly higher (*P* < 0.0005) compared to healthy controls (0.205; 0.00–0.80). Serum concentration of SCL 70 in the SLE patients (1.10; 0.71–3.33) was significantly higher (*P* < 0.0005) compared to healthy controls (0.155; 0.00–0.28) (Table 3).

**Figure 8:**
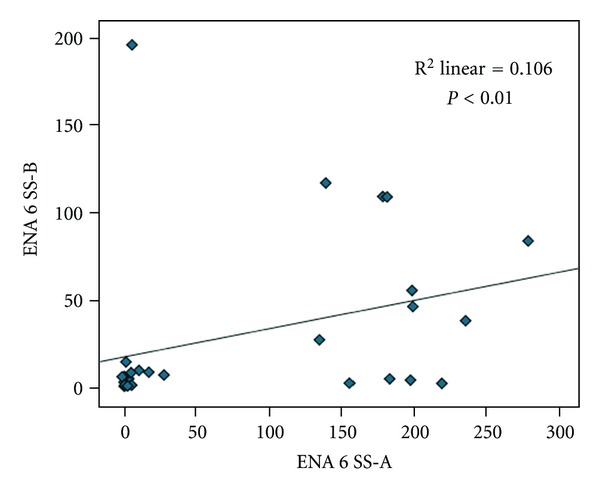
Spearman's correlation analysis between ENA6 SS-A and ENA6 SS-B.

**Figure 9:**
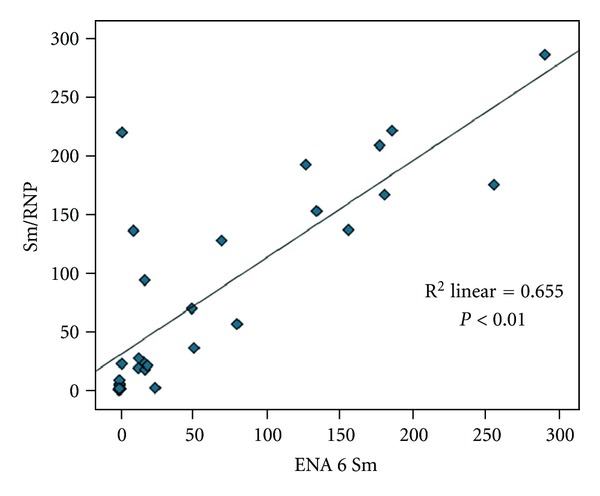
Spearman's correlation analysis between ENA6 Sm and Sm/RNP.

**Figure 10:**
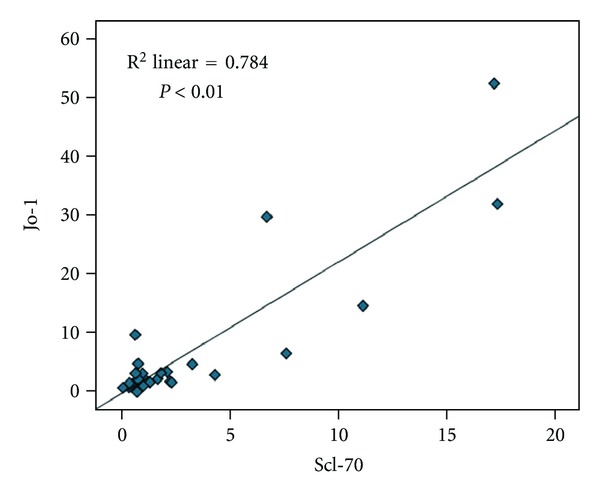
Spearman's correlation analysis between SCL 70 and Jo-1.

Results have shown significant correlation between ENA6 SS-A and ENA6 SS-B (*r* = 0.99; *P* < 0.01) (Figure 8.); ENA6 Sm and Sm/RNP (*r* = 0.801; *P* < 0.01) (Figure 9); Jo-1 and SCL 70 (*r* = 0.72; *P* < 0.01) (Figure 10). Results did not show significant correlation between other markers of ENA6 profile. 

**Figure 11:**
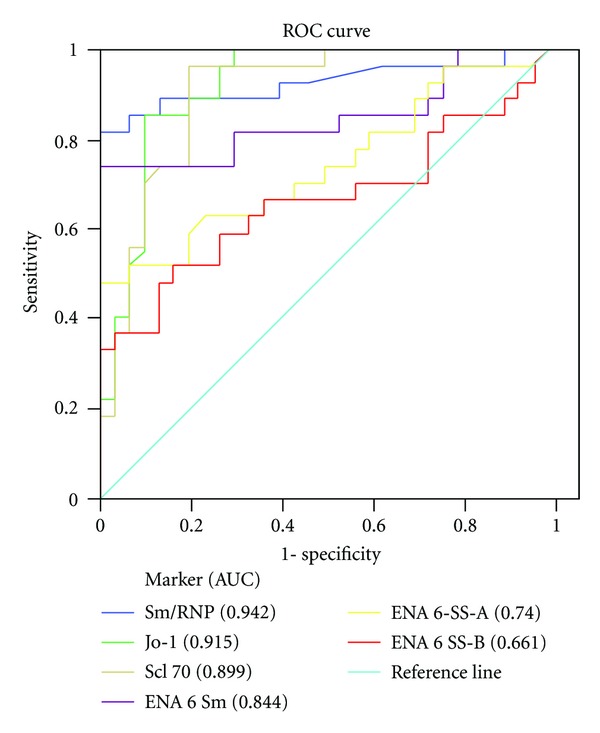
Receiver operating characteristic (ROC) curve of ENA6 profile markers for differentiation between SLE for patients and healthy control.

The ROC curves for ENA6 SS-A, ENA6 SS-B, ENA6 Sm, Sm/RNP, Jo-1, and SCL 70 in the patients with SLE and healthy controls are shown in Figure 11. 

In our research according to calculations from ROC curves, Sm/RNP is clearly very important marker for diagnosis of SLE (cut off ≥ 9,56 EU; AUC 0,942). Unexpectedly, the first that follows is Jo-1 (AUC 0,915); then Scl-70 (AUC 0,899); Sm (AUC 0,844); SS-A (AUC 0,740); and SS-B (AUC 0,661).

Based on the proposed cut-off values, the sensitivity, and specificity of the ENA6 markers were calculated.  Table 4 shows the predictive power of each markers in distinguishing patients with SLE and healthy controls. 

The percentages of patients that had elevated concentration level of ENA antigens were as follows: Sm,/RNP, Sm, SS-A, SS-B, Scl-70 and Jo-1; 73,3%; 66,6%; 50%; 40%; 10%; 6,6%, respectively.

## 4. Discussion and Conclusion

 The aim of this study was to evaluate concentration values of each antibody of ENA-6 profile in SLE and to determine concentration values of CIC and Sm-antibody as potential immunobiological markers in SLE. Furthermore, we aimed to establish whether there is a correlation between Smith antibody in sera and levels of CIC and disease activity [36].

Our results have shown that most valuable marker for SLE activity monitoring is Sm/RNP, than followes Jo-1. Obtained results are not in the accordance with the reports from other authors [37–39]. Possible explanations for our results might be due to small study sample. 

High titer of anti-Sm antibody is highly SLE specific although low-titer anti-Sm in ELISA has been reported in other diseases [40]. Anti-Sm antibodies are in fact found without RNP because both proteins associate with common snRNA [40]. 

 For six antigens that comprise ENA-6 profile used in our study (SS-A, SS-B, Sm, RNP, Scl-70, and Jo1) we reported concordances, sensitivity, and specificity in range of 60 to 100%. The highest specificity has been reported in Sm/RNP and Sm (100%); while the highest sensitivity has been reported at Scl-70 (96%) and Jo 1 (83%). There is evidence about correlation among Scl-70 and Jo1; as well as among Sm and Sm/RNP; among SS-A and SS-B; while there is no correlation among those three couples of antibodies [41]. Our results on concentration level and sensitivity of Scl-70 antibody is not in the accordance with other reports [42].

 High incidence of Scl-70 (10%) reactivity suggests that ELISA monitoring of this antibody produces more false positive results than other multiplex assay [42, 43]. In our research according to calculations from ROC curves, Sm/RNP is clearly a very important marker for diagnosis of SLE. Surprisingl and interestingly first that follows is Jo-1.

ELISA monitoring of extractible antinuclear antibodies—Sm and CIC made, it possible to identify characteristic changes in serum specimens that are significantly in correlation with disease activity in patients with SLE [43]. Despite some reported prospective studies that suggest no correlation of those immunomarkers and lupus flares and disease activity in this study, we noticed the correlation between Sm antibody as well as CIC and disease activity. It is of note that those two did not show significant correlation among themselves [44–48]. 97% of patients had a positive ANA antibody testing that is usual in some other researches. Anti-dsDNA is less sensitive but more specific for SLE diagnosis [44, 45]. They can be found in the sera of 55% to 80% patients with SLE but not in the sera of the healthy controls which is also confirmed in our study. Most of the investigators indicate that anti-dsDNA antibodies are useful markers of SLE overall activity of SLE [44].

An antibody to Sm, a ribonucleoprotein found in the nucleus of a cell, is almost exclusively present in people with lupus. It is present in 20% of people with the disease but it is rarely found in people with other rheumatic diseases and its incidence in healthy individuals is less than 1%. Therefore, it can be helpful in confirming the diagnosis of systemic lupus [49–56]. Table 2 shows optimal cut-offs, with high confidence interval, sensitivity of 70%, and specificity of 100%.

Detecting ANA, Op De Beéck et al. compared the results obtained using indirect immunofluorescence (IIF) and BioPlex 2200, and discovered that BioPlex test result interval-specific likelihood ratios increased with increasing antibody concentration. BioPlex provided presence of at least three antibodies simultaneously. They concluded that test result specific likelihood ratios and the presence of multiple autoantibodies help with the interpretation of data generated by multiplex immunoassays [57].

Circulating immune complexes are present in many individuals with SLE and rheumatoid arthritis (RA), especially in those with any of the vasculitis complications. Levels of CICs have been reported to show correlation with disease activity, especially during active phases of the disease [45–48, 56]. In this study, results from ROC curve are suggesting that CIC might be even better marker for SLE activity than Smith antigen. 

Our results have revealed that all of the used biomarkers do accompany the diagnosis of active SLE but their use as a solo marker does not allow classification of SLE patients. Furthermore, it can be concluded that laboratory tests are no more effective than clinical examinations for detecting disease relapse, but are helpful for confirming the activity of SLE.

## Figures and Tables

**Table 1:** 

Antibodies of ENA-6	*μ*g/mL	CIC	*μ*g/mL
Negative	<10	Negative	<40
Equivocal	10–15	Equivocal	40–50
Positive	>15	Positive	>50

**Table 2:** Optimal cut-off, area under the curve with 95% confidence interval (AUC, 95% CI), sensitivity, specificity, positive and negative predictive value of ENA6, SM, and CIC in differencing between SLE patients and healthy control.

SLE patients versus healthy control						
Marker	Optimal cut-off	AUC (95% CI)	Sensitivity (%)	Specificity (%)	Positive predictive value (%)	Negative predictive value (%)
ENA6 Sm	≥9,56 EU	0,809 (0,690–0,928)	70%	100%	100%	76%
CIC	≥54,24 EU	0,931 (0,854–1,00)	76%	100%	100%	81%

SLE: systemic lupus erythematosus; AUC: area under the curve; CI: confidence interval; ENA6 Sm: extractable nuclear antigens 6 Sm; CIC: circulating immune complexes; EU: elisa units.

**Table 3:** The median and interquartile range of serum concentration of ENA6 profile in the healthy subjects and in the SLE patients.

Marker	Status	Percentiles			
		25	50	75	*P* values
ENA 6 SS-A	Healthy controls	1.20	3.55	5.75	*P* < 0.001
	SLE patients	2.85	11.70	183.51	
ENA 6 SS-B	Healthy controls	1.45	3.10	5.90	*P* < 0.01
	SLE patients	1.71	6.64	46.32	
ENA 6 Sm	Healthy controls	0.60	1.65	2.625	*P* < 0.0005
	SLE patients	2.27	19.93	135.95	
Sm/RNP	Healthy controls	0.50	1.20	2.80	*P* < 0.0005
	SLE patients	17.70	56.61	166.96	
Jo-1	Healthy controls	0.00	0.205	0.80	*P* < 0.0005
	SLE patients	1.40	2.22	4.79	
SCL 70	Healthy controls	0.00	0.155	0.28	*P* < 0.0005
	SLE patients	0.71	1.10	3.33	

**Table 4:** Optimal cut-off, sensitivity, specificity, positive and negative predictive value of ENA6 SM, and CIC in between SLE patients and healthy control.

SLE patients versus healthy control					
Marker	Optimal cut-off	Sensitivity (%)	Specificity (%)	Positive predictive value (%)	Negative predictive value (%)
Sm/RNP	≥8,33 EU	75%	100%	100%	81%
Jo-1	≥1,005 EU	83%	90%	89%	84%
SCL-70	≥0,385 EU	96%	80%	83%	96%
ENA6 Sm	≥9,56 EU	70%	100%	100%	76%
ENA6 SS-A	≥5,90 EU	64%	76%	72%	69%
ENA6 SS-B	≥5,44 EU	60%	73%	69%	65%

SLE: systemic lupus erythematosus; EU: elisa units.
